# “Such an institution represents the circle of life” – bringing an inpatient hospice into an academic setting: a pre-implementation exploratory study

**DOI:** 10.1186/s12904-023-01220-6

**Published:** 2023-07-19

**Authors:** Kim Dillen, Thomas Montag, Birgit Weihrauch, Heidrun Golla, Raymond Voltz, Julia Strupp

**Affiliations:** 1 Department of Palliative Medicine, Faculty of Medicine and University Hospital, University of Cologne, Kerpener Strasse 62, 50937 Cologne, Germany; 2 “Endlich. Palliativ & Hospiz” Association, Cologne, Germany; 3 Center for Integrated Oncology Aachen Bonn Cologne Duesseldorf (CIO ABCD), Faculty of Medicine and University Hospital, University of Cologne, Cologne, Germany; 4 Center for Health Services Research, Faculty of Medicine and University Hospital, University of Cologne, Cologne, Germany

**Keywords:** Palliative medicine, Hospice, End-of-life care, Prospective study, Qualitative research

## Abstract

**Background:**

To combine the benefits of hospice and palliative care, the integration of both seems self-evident. Aim of this study was to explore clinical staff’s and volunteers’ expectations and concerns of the first university hospice in Germany planning for implementation.

**Methods:**

Staff and volunteers of the Department of Palliative Medicine of the University Hospital in Cologne received questionnaires and were interviewed following three themes of interest: opportunities, challenges, general criteria. Questionnaire results were analyzed descriptively using mean ± SD and percentages, open-ended questions and interviews were analysed using content analysis.

**Results:**

A total of 28/100 questionnaires was returned (*n* = 17 clinical staff, *n* = 11 volunteers) and 18 interviews conducted. The majority of both clinical staff and volunteers estimated the need for a university inpatient hospice as rather to very high (64.7% and 81.8%, respectively). Our findings revealed that most clinical staff and volunteers anticipated improvements with the intended university inpatient hospice, although their expectations were divided between both hope and concern while adhering to legal and general requirements, which they feared might oppose such a project. Participants expressed concern about leadership and staffing plans, albeit most pronounced among clinical staff. Nursing staff repeatedly articulated concerns about being interchanged between the palliative care ward and the intended inpatient hospice while they had explicitly chosen to work in palliative medicine.

**Conclusions:**

The overall high level of anticipated progress and excitement is very encouraging. Albeit serious concerns were mentioned, our results indicate that all participants believe in a positive impact and highlight the need of developing a solid concept.

In order to implement such a hospice within a university setting, it is important to consider multilevel contextual factors such as system-level factors (funding, external and internal regulations), organization-level factors (leadership, staff motivation), and patient-level factors (adaptability to patients' needs). Our findings illustrate the importance of understanding the context of practice before implementation. Our pre-implementation study helps identify critical views from staff members and volunteers that may hinder or advance the implementation.

**Trial registration:**

The study was registered at the German Clinical Trials Register (#DRKS00021258) on April 17^th^ 2020.

**Supplementary Information:**

The online version contains supplementary material available at 10.1186/s12904-023-01220-6.

## Contributions to the literature

- The goal of this pre-implementation study was to gather from various perspectives how the basic idea of integrated hospice and palliative care can be realized within a university hospital in order to implement the first academic hospice in Germany.
- This study identified barriers and facilitators before implementing the first hospice within a university setting.
- Although several concerns have been raised, our results indicate that all participants believe in a positive impact and highlight the need of developing a solid concept integrating multilevel contextual factors.

## Background

Hospice care focuses on the quality of life and comfort of a person who is facing the end of life and on their family caregivers [9, 20, 21]. In Germany, inpatient hospices are independent free-standing institutions offering individual support for the dying adult including nursing, symptom relief, grief support, as well as social, spiritual, and homelike aspects [9, 20, 21]. In fact, international studies have shown significant improvements in family caregiver well-being, bereavement outcomes and patient, family, and physician satisfaction [11, 17]. However, a fair comparison on an international level is hampered by the different concepts of palliative and hospice care. In Germany, hospices have a different mission than palliative care units which are usually integrated into a hospital and aim at medical stabilization with respect to complex symptom treatment [20, 21]. In other countries, these concepts are often used interchangeably. We will focus on the concepts used in the German healthcare context for the remainder of this paper.

To combine the benefits of both inpatient hospices and palliative medicine at a university hospital, i.e., humanitarian aspects and high-tech medicine, the integration of both seems self-evident. These affiliations allow university hospitals to further end-of-life care by expanding its continuum of care. Palliative care patients could benefit from such a unit, as it can be challenging to transfer palliative care patients after medical stabilization in due time to a hospice so this makes for an easy, timely, and seamless transition. To the best of our knowledge, there are only five university inpatient hospices worldwide. Jegier et al. [11] evaluated one of them and found longer hospice lengths of stays and lower hospital costs [11]. This is also true for Germany as a stay at a hospice is less expensive for insurance companies than at a palliative care unit.

However, these joint units also raise several concerns that need to be addressed. First, it is of utmost importance to understand the concepts of a hospital, palliative care unit and a hospice. It seems particularly challenging to interweave them as a palliative care unit at a hospital primarily focuses on anticipating, preventing and treating symptoms experienced by seriously ill patients while the perspective of the volunteer hospice civic movement might fade into the background. Second, patients admitted to a university hospital often have a more acute symptomology and greater needs for pain and symptom management, while people in a hospice need qualified nursing/psychosocial care and support with dying [9, 20, 21]. This relates to another essential difference, which is the length of stay. Palliative care patients are usually discharged after symptom relief or are referred to a hospice where they will most likely live out their remaining days [21]. Third, there are different architectural specifications, i.e., palliative care units as part of a hospital are subject to hospital regulations, while inpatient hospices are subject to the home minimum building code and have an autonomous care mandate. The interior design of an inpatient hospice must be adapted to the needs of its residents and have a homelike character [9, 21]. Last, economical and funding differences play a major role. While full funding is expected for a palliative care unit, partial funding with compensation through donations is required by the legislature for an inpatient hospice [9].

To explore clinical staff’s and volunteers’ expectations and concerns of the first university inpatient hospice in Germany, we conducted semi-structured interviews and handed out short questionnaires at the Department of Palliative Medicine of the University Hospital in Cologne, Germany. These findings will contribute to the development of a suitable concept prior to the implementation of the planned unit. To the best of our knowledge, an inpatient university hospice does not yet exist in Germany and this is the first prospective qualitative study of such a unit.

## Methods

### Setting

The study was conducted at the Department of Palliative Medicine of the University Hospital of Cologne, Germany. All participants gave their written informed consent. The study was approved by the local ethics committee (reference No. 20–1011), registered at the German Clinical Trials Register (#DRKS00021258), and conducted in accordance with the declaration of Helsinki [8].

All professionals and volunteers—*n* = 100 in total—received a one-page questionnaire. This was to ensure that the views and perspectives of all participants were taken into account. The study materials were enclosed with an invitation letter and the survey would take approximately 10–15 min to complete.

### Design and data collection

This was a pre-implementation exploratory study using a combination of open-ended survey questions and semi-structured in-depth interviews. We had originally planned focus groups. Due to the COVID-19 pandemic that had then started, we were prohibited from conducting focus groups and participants were even more limited in their resources in terms of time. The decision was then made for individual interviews via video conference that were easier to plan and conduct. After the ethics committee had approved the amendments, data collection could begin.

Step 1: The survey contained a self-developed questionnaire including six open-ended questions about possible i) opportunities, expectations, ii) challenges, concerns, and iii) criteria, general requirements. The questionnaire commenced with a closed-ended question concerning the need for a university inpatient hospice on a five-point Likert scale and concluded with an open-ended question for additional remarks. The self-developed questions were informed by discussions with clinical experts, health services researchers as well as by our research objectives and reviewed literature, and co-developed with the head of the working group “hospice” at the Department of Palliative Medicine (TM). It was then distributed to all division heads of the Department of Palliative Medicine who passed our information material to their staff.

Step 2: The interview guide was developed by KD (psychologist and PhD in health sciences) and JS (social and behavioural scientist and PhD in health sciences). The interview guide (supplemental file 1) was derived from the study-specific questionnaire allowing interviewees to clarify insights and elaborate on all sections of the questionnaire in more detail, providing a degree of freedom and adaptability while covering the same areas of information [14]. To enhance credibility, the topic guide was pre-tested and discussed with research and clinical staff within a research workshop (conducted online).

All interviews were recorded digitally and performed by the first author (KD). JS familiarised herself with a random sample of the responses. Afterwards, the codes and categories were discussed and negotiated between the authors and adjustments were made if necessary. Verbatim transcription was done by a professional transcriber who had signed a confidentiality agreement. Demographical information was collected at the beginning of each interview keeping the information to a minimum to ensure the greatest level of anonymity. The survey was conducted anonymously. In this respect, we do not know who participated in both (survey and interview) or only in one of the two.

### Participants

Clinical staff and volunteers had to be affiliated with the Department of Palliative Medicine of the University Hospital of Cologne, Germany, needed to be 18 years of age or older and able to give informed consent. Thus, included in the data collection were nurses, physicians, psychologists, case manager, social worker, chaplains, and volunteers.

### Data analysis

The questions concerning the need for a university inpatient hospice were analyzed descriptively, while open-ended questions were analyzed and categorized based on content by the first author (KD), both using SPSS software (v.25, SPSS, Inc., Chicago, IL). All responses from the open-ended questions were first listed on a coding sheet to create tentative and broad-based categories. They were then sorted into meaningful categories and sub-categories and finally quantified using frequency counts to understand the underlying contextual use of the content [10]. These were then compared descriptively between responses from clinical staff and volunteers. Statements to the last question for additional remarks concerned similar issues to the other questions so they were categorized together.

Transcribed interviews were analyzed similarly using inductive qualitative content analysis [7, 15]. First, open coding headings were created inductively from the material corresponding to the three sections asked in the interview [12]. These were then further explored, revised, and combined with similar content within a feedback loop and finally reduced to main and sub-categories [7, 12]. All coded segments were then compared for differences and similarities and summarised in a multi-stage process. The categorization of ambiguous text segments was discussed with the last author (JS) until a consensus was found and optimized within a peer debriefing. Finally, the frequencies of all coded frequencies were analyzed [7]. Meaningful quotes were added.

As this study used a combination of a questionnaire and in-depth interviews, the responses from the questionnaire were analyzed first to get a better and in-depth understanding of the results. Themes emerging from the questionnaire and interviews were clustered according to common categories. Distinctive categories were also identified.

Data were analyzed using MAXQDA 18 software [16]. Descriptive and frequency statistics were calculated using SPSS software (v.25, SPSS, Inc., Chicago, IL).

## Results

### Need for hospice

A total of 28/100 questionnaires was returned (*n* = 17 clinical staff, *n* = 11 volunteers). The majority of both clinical staff and volunteers estimated the need for a university inpatient hospice as rather to very high (64.7% and 81.8%, respectively) (see Table 1).

**Table 1:** Need for hospice

Questionnaires	Clinical staff *n* = 17	Volunteers *n* = 11
Need for hospice (n, %)		
Very high	5, 29.4	7, 63.6
Rather high	6, 35.3	2, 18.2
Rather low	4, 23.5	0, 0
Very low	1, 5.9	0, 0
Don’t know	1, 5.9	2, 18.2

### Demographical characteristics of interviewees

We conducted twelve interviews with clinical staff and six interviews with volunteers. Characteristics of the sample can be found in Table 2.

**Table 2:** Demographic data of interviewees

Interviews	Clinical staff *n* = 12	Volunteers *n* = 6
Sex (m) (n, %)	6, 50.0	1, 16.7
Age (M ± SD)	47.5 ± 3.3	50.8 ± 3.9
Work experience (in years) (n, %)		
< 5	3, 25.0	5, 83.3
5–10	2, 16.7	1, 16.7
11–15	2, 16.7	0, 0
16–20	1, 8.3	0, 0
> 20	4, 33.3	0, 0

### Qualitative analysis

Results from questionnaires and in-depth interviews were analyzed separately, however, as expected there was great overlap so common categories and sub-categories were merged. Within each of the three blocks, three categories were identified: staff, organizational level, patient-centered care each with underlying sub-categories (Table 3).

**Table 3:** Categories and sub-categories from questionnaires and interviews

Categories and sub-categories	Clinical staff		Volunteers	
	**Questionnaires**	**Interviews**	**Questionnaires**	**Interviews**
**1. Opportunities and expectations (**number of segments that were assigned to this particular code)				
*a. Staff*				
Staffing	0	25	2	21
Leadership^a^	0	2	0	0
Number of statements regarding staff (n, %)	0, 0.0	27, 16.56	2, 3.70	21, 15.22
*b. Organizational level*				
Innovation	9	18	3	4
Expanding and intensifying competencies and services	9	9	10	18
Relieving existing resources	1	3	0	2
Teaching and research	4	7	1	2
Strengthening synergies and legal requirements	22	17	10	12
Equitable access	3	8	2	4
Public relations	5	5	7	12
Food service^a^	0	5	0	4
Autonomy of hospice / independence from palliative care unit	8	8	0	1
Structural factors / hospice character	9	41	1	41
Number of statements regarding the organizational level (n, %)	70, 81.40	121, 74.23	34, 62.96	100, 72.46
*c. Patient-centered care*				
Continuity of care	16	6	13	4
Medical care	0	4	5	2
Number of statements regarding patient-centered care (n, %)	16, 18.60	15, 9.20	18, 33.33	17, 12.32
**2. Challenges and concerns**				
*a. Staff*				
Staffing	5	12	5	1
Leadership	1	3	0	0
Number of statements regarding staff (n, %)	6, 11.76	15, 19.74	5, 15.15	1, 8.33
*b. Organizational level*				
Bureaucracy	8	16	1	0
Financing	2	9	4	4
Rejection of hospice movement	1	2	0	0
Neglecting existing structures	2	1	0	0
Strengthening synergies and legal requirements	3	16	1	1
Teaching and research^b^	5	0	2	0
Autonomy of hospice / independence from palliative care unit	10	5	2	0
Structural factors / hospice character	7	2	7	6
Number of statements regarding the organizational level (n, %)	38, 74.51	51, 67.11	17, 51.52	11, 91.67
*c. Patient-centered care*				
Patient understanding	2	8	1	0
Number of statements regarding patient-centered care (n, %)	2, 3.92	8, 10.53	1, 3.03	0, 0.00
**3. Criteria and general requirements**				
*a. Staff*				
Staffing	23	29	8	5
Leadership	1	4	1	0
Number of statements regarding staff (n, %)	24, 23.76	33, 40.24	8, 12.31	5, 27.78
*b. Organizational level*				
Concept	9	8	4	1
Financing	4	6	0	0
Strengthening synergies and legal requirements	4	7	0	0
Teaching and research^b^	1	0	0	0
Public relations^a^	0	0	0	1
Food service^a^	0	2	0	0
Autonomy of hospice / independence from palliative care unit	9	2	2	0
Structural factors / hospice character	30	23	16	8
Number of statements regarding the organizational level (n, %)	57, 56.44	48, 58.54	22, 33.85	10, 55.56
*c. Patient-centered care*				
Humanity	3	1	9	3
Number of statements regarding patient-centered care (n, %)	3, 2.97	1, 1.22	9, 13.85	3, 16.67

^a^ was mentioned in interviews only

^b^ was mentioned in questionnaires only

#### Staff

Responses from both clinical staff and volunteers were pending between opportunities, challenges, and general criteria in relation to what they expected for leadership and staffing. Both were slightly more important for clinical staff than for volunteers. They expected the leadership position to be filled by nursing staff but at the same time feared that this might not be possible.

For example, it must be led by nurses, i.e., the management position must be filled by a nurse and therewith be autonomous from other sub-units of the department which are led by a medical director. (clinical staff, 01)

There was a general consensus that the team should consist of an interdisciplinary team who must all communicate interprofessionally. Important aspects mentioned were the nursing staff-to-patient ratio and the recruitment of highly qualified staff who are intrinsically motivated to work in a hospice. To keep staff motivated, regular meetings and updates with full transparency and involvement of all employees, already in the planning and development phase, was considered essential. The biggest challenges forecast were the interchangeability of staff and the different financing of both.

There must be a clear separation of staff, that is, that new employees are hired who want to work in the hospice and then only work there and that those who currently work in the palliative care unit do not have to leave and work in the hospice. That would certainly work for a day, but not for the long term. I don’t even know if that’s allowed by the authorities. (clinical staff, 04)

#### Organizational level

Clinical staff in particular expressed a genuine sense of apprehension for the integration of both concepts. This cooperative project was considered a revolutionary, pioneering, and innovative establishment in the palliative-hospice setting following the English model where hospices provide palliative and end-of-life care, thus overcoming the different sectors by integrating all levels of medical, therapeutical, and spiritual care. Concurrently, provided appropriate concepts are available, it might open the doors for people other than oncological patients typically treated in palliative care.

Well, in my opinion, the greatest opportunity is the fact that we could include groups of patients into our care, if we wanted to - of course, an appropriate concept would have to be developed for this - which we do not care for in a prototypical hospice. I am thinking primarily of severely affected neurological patients such as patients with amyotrophical lateral sclerosis or multiple sclerosis or Parkinson's disease or whatever. (clinical staff, 03)

Both clinical staff but more volunteers expected an expansion and intensification of competencies and services. This included more volunteer work and co-therapies, more opportunities for patients and family caregivers to mingle as well as individual end-of-life care.

I even believe that it feels very good for relatives to get in touch with one another. I think that's a good way to start a conversation and start mourning, simply put - you're not alone. (volunteer, 09)

Especially clinical staff spoke of a great opportunity to relieve existing resources as there are not enough hospices in the area and to further the current use of research methods. They also felt that the development and implementation of teaching practices that advance students’ knowledge and prepare them for working with terminally ill people was essential. Both teaching and research were mentioned in all three blocks with clinical staff, in particular, being worried that resources and money will only be invested in research and patients’ needs to converse might be exploited for research purposes.

In addition, they emphasized the strengthening of synergies as an outstanding opportunity, general criterion but also challenge. The close cooperation with the Department of Palliative Medicine, including its sub-units, and other departments is desired.

… and certainly that there is no negative competition between the different departments of the university clinic but that the expertise and scientific knowledge of every department will be accessible to hospice residents. (volunteer, 15)

Herein, the integration with the university clinic as a large organization was discussed heavily. On the one hand, this would allow for faster access to medical doctors as well as less bureaucracy and paperwork. On the other hand, this would mean less decision-making and flexibility for employees and too much medical/palliative care. It was stressed that a hospice has different legal and monetary requirements whereas the university clinic as large, powerful organization must bring profit and provide the maximum level of curative medical care.

Then, of course, financing must be completely separate. On the one hand, we are part of a hospital and must generate a profit. On the other hand, an inpatient hospice is not allowed to make a profit but depends on donations. (clinical staff, 06)

Although there was a general consensus for a cooperative concept integrating a hospice into a university clinic, it was feared that current existing structures would be neglected in favor of the university hospice. Both groups hoped that the new inpatient university hospice would result in equal access to medical and therapeutical care without budgeting limits. Similarly, they hoped for equitable access for everyone, specifically for people who usually get lost in the health system.

… but I think it's important to give people access- access to people who don't have access to palliative care yet. (clinical staff, 01)

Both groups wished for and considered it an urgent requirement to publicize and normalize death within society, which is an important aspect in the current pandemic, and suggested using this concept as stigma-change intervention for reduction of negative attitudes associated with a hospice.

I think these topics [grief, death, and dying] will become more present at university but also in society, this project might raise people’s interest in these topics, reducing their fear of death, while decreasing the taboo element of death. (clinical staff, 11)

Clinical staff felt that freshly prepared food adapted to the individual by a professional cook was essential as this is often one of the patients’ last wishes and only highlight left.

And for many patients who can still eat, that is the only highlight left, a delicious dish. And I would find it nice (…), that there is someone permanently employed, a cook, who perhaps cannot respond to every wish, but can respond to wishes and can prepare a delicious meal (…) because as I said, that is the last true feeling of joy for many. (volunteer, 09)

Alternatively, it should be allowed for relatives to cook and dine together as this relates to a better quality of life.

They also criticized the bureaucratic cumbersome nature and disproportionately long decision-making processes within a university hospital. The project has to be bound by collective agreements and comply with legal requirements taking into account the responsibilities of the authorities. Another important aspect related to the budget plan. This included cost coverage, funds being cut or frozen, financial restrictions on the length of stay, profitability, and different financing for both institutions.

(…) only the financial aspect, that patients do not care for. That the palliative care ward is financed by the health insurance and the hospice is financed by the health insurance and long-term care insurance, that of course, is of very little interest, very little. (clinical staff, 13)

Concurrently, clinical staff expected sufficient budgeting to fulfill patients’ last wishes and for sustainable staff management. A few employees also mentioned the possible rejection of the hospice movement as general hurdle.

Another fear is, on the other side, what I have just mentioned, that the hospice movement is like "For God's sake, now the university medicine also wants to take possession of the hospice movement” and might therefore reject it completely without thinking about it any further. (clinical staff, 06)

Last, especially clinical staff stressed the importance of developing a solid, innovative, and progressive concept. The difference between a palliative care unit, a regular and a university hospice must be defined and clear structures should prevail. This should be determined before the localities and architecture are set and with the involvement of all employees. In that regard, clinical staff, in particular, hoped for an autonomous, independent hospice. They expected it to be a separate sub-unit of the palliative care center. Similarly, the hospice character had to be ensured, both conceptually and structurally. The focus must be on care and supportive therapies with a calm, peaceful atmosphere.

I'm a bit afraid that it will be too medical and not cozy enough, which usually characterizes a hospice, more peace and quiet, little hustle and bustle, that it's just like home. (clinical staff, 07)

Structurally, the most important aspect mentioned was the spatial design, i.e., the optimal architecture. They hoped for single, spacious rooms with a window and access to a garden, overnight accommodations for relatives, recreation rooms, a farewell room or "room of silence” and lounges to connect with one another. The predominant focus was a protected garden with barrier-free access. However, they were well aware, that this warranted major challenges and hurdles.

#### Patient-centered care

Both groups expected a more holistic, continuous approach for patients, allowing for quicker and easier patient transfer concurrently respecting patients’ wishes to stay in one location. This would close existing gaps of care, in particular with respect to budgeting for medical doctors not having to write referrals and patients not having to worry about being admitted to a hospice.

And that is a holistic approach, to say that the combination of palliative medicine and hospice allow for a qualitative improvement at the end of life, I think that is extremely important for patients and their relatives because it is stressing them out, once they have gotten used to the concept of palliative care having to leave and where to receive hospice care. (clinical staff, 11)

Volunteers also stated that optimal and expeditious medical care for all patients is imperative. However, clinical staff worried that the ease of transfer might be moving too fast towards death for patients. Their main concern was to separate staff and rooms so patients would understand whether they are palliative care patients or residents of a hospice.

Let's assume that we refer a patient from another department to the palliative care ward (…) if it were not spatially separate from the hospice, then I wonder whether that patient understands (…), feels, sees that this [hospice] is yet another place. (clinical staff, 12)

Volunteers further felt that human rights and principles have to be guaranteed. Hospice residents, just like any palliative care and other patients, must be given the chance to die in dignity and peace, with professional support and the greatest possible self-determination. Aspects of humanity and spirituality were emphasized as framework conditions.

Humanity should really be the highest priority, humanity and real dignity, which is being said over and over again, yes, one should - everyone wishes to die in dignity. (volunteer, 15)

For supporting quotes please see supplemental file 2.

## Discussion

To the best of our knowledge, there is no university inpatient hospice in Germany yet, so this is the first prospective qualitative study of such a unit. This study deliberately focused on the perspective of clinical staff and volunteers who currently work at the Department of Palliative Medicine of the University Hospital in Cologne, Germany, and will most likely play a major role in the proposed university inpatient hospice.

Our findings revealed that most clinical staff and volunteers anticipated improvements with the intended university inpatient hospice, although their expectations were divided between both hope and concern while adhering to legal and general requirements, which they feared might oppose such a project. Participants expressed concern about leadership and staffing plans, albeit most pronounced among clinical staff. It was noticeable how especially nursing staff repeatedly articulated concerns about being interchanged between the palliative care ward and the intended inpatient hospice while they had explicitly chosen to work in palliative medicine. Caring for complex and terminally ill patients and their principal caregivers has been associated with work- and client-related stress [22, 23], which is highly pronounced in a hospice [22] and might be one reason for the above mentioned fear. It is therefore advisable that superiors grant their employees a reliable field of application at work as well as the opportunity to restitute and self-care to keep their level of stress to a minimum and allow for a healthy work-life balance. As this is insufficiently guaranteed, the vast majority of clinical staff works part-time resulting in the well-known workforce shortage of certified professionals in palliative care. As the number of hospice programs increases, this leads to a mismatch between workforce capacity and clinical need [17]. A second fear pending between expectations and general requirements related to staff was the leadership position. According to the legal requirement of a regular hospice in Germany, the leadership position has to be filled by nursing staff [9]. The intent is therefore to have nursing staff in the leadership role. However, participants might not have been fully aware of this since they expressed concern due to the affiliation to the palliative care ward, which is led by a medical director. It would be helpful to match expectations, hopes, and concerns of clinical staff and volunteers with the rules and regulations that such a project is bound to in regular meetings and ensure a transparent communication.

Clinical staff expressed a clear need for teaching and research, which is not surprising given its attachment with the university hospital. Research in the intended university hospice is of particular importance as there is rarely any hospice research in Germany. It should also be a high priority for patients and hospice residents giving them time with and attention from researchers [1, 3, 25]. This, however, concurrently raised serious ethical concerns as to not abuse residents’ need to converse and that dying people are too vulnerable to participate in research [5]. Research and our own experience, however, have shown that most hospice residents, palliative care patients and their relatives involved in research found the experience helpful and beneficial and value the opportunity to choose to participate [2, 24]. Similarly, a difference was found in the description of strengthening synergies between sub-units of the Department of Palliative Medicine and other departments of the university clinic and the concern that the university hospice would have an academic medical character rather than a hospice character. Therefore, a solid, well-designed, and innovative concept is of particular importance. Change always takes time and is a complex, multi-dimensional process with a purpose. It can be supported or restrained by expectations, knowledge, or level of planning. In our study, clinical staff and volunteers described expectations, hopes, and concerns that can be interpreted as both supporting and restraining factors. By involving clinical staff and volunteers into this study, we might have reduced the level of concern already.

In Germany, 95% of costs for an inpatient hospice stay are borne by the statutory health insurance and long-term care insurance while the remaining 5% are borne by donations [9]. Conversely, a stay in a palliative care ward, just like a stay in any other hospital wards, is financed entirely by the statutory health insurance. This difference raised serious concerns about staff and budget planning as well as patients’ length of stay. The average length of stay in a regular inpatient hospice is usually between 3–4 weeks with 90% of patients dying there while around 70% of patients die in a palliative care ward having stayed around 12–14 days [6]. While previous research found no difference in length of stay in a hospice between patients referred from academic and non-academic medical centers [4], these issues need to be further explored within the scope of the concept development of the intended university inpatient hospice. Importantly, however, the fundamental difference in financing, i.e., the fact that a small percentage of a hospice is financed by donations, suggests a pronounced relation to the regional population and outreach to citizenry. For society, this project can potentially be used to publicize death and help us prepare for bidding farewell and dealing with serious illnesses which seems of special importance given the fact that we are currently surrounded by dying, fatalities, and ensuing fears as a result of the pandemic. Thinking of volunteers as essential to any hospice for extending reach and impact [9], this project might increase the recruitment of volunteers.

Another important finding was that although the general consensus is that hospice residents often experience loss of hunger as the dying process unfolds [19], interviewees highlighted that maximizing food service by providing nutritious food for terminally ill patients with compromised appetites and eating capacities will increase their quality of life [18]. They felt that patients, their caregivers, and staff participating in adequate food preparation and gathering at mealtime was essential. This would allow families to connect and interact with staff, creating a homelike, supportive environment. Providing enjoyable drinks and food, access to a garden, sitting at the bedside of a dying patient contributes to the best possible palliative and hospice care [13].

### Strengths and limitations

This was an explorative, pre-implementation mixed-methods study, which requires the inclusion of all possible participants. In this study, information material was distributed to all division heads of the Department of Palliative Medicine who have passed it to their staff. About 2/3 of all employees did not partake in the study, possibly due to time constraints, lack of interest or positive attitude towards the intended university inpatient hospice. However, we explicitly asked about foreseen challenges and hurdles, so negative statements were welcomed and, in fact, expressed by participants. Due to the low number of participants who originated from the Department of Palliative Medicine of the University Hospital of Cologne only, the answers and especially the closed-ended question “need of a hospice” has to be interpreted with caution. This study is exploring healthcare professionals’ and volunteers’ perspectives who currently work in the Department of Palliative Medicine. We have therewith ensured the greatest level of heterogeneity with our sampling method, thus reducing bias, which we consider a strength, especially since sex and age were normally distributed. This does, however, clearly limit the generalizability. Therefore, this study should be followed by another qualitative study from the perspectives of palliative care patients, hospice residents, and their family caregivers as well as healthcare professionals from other departments of the university hospital Cologne, palliative care units, and hospices.

To indicate trustworthiness of our results and enhance readability, representative quotations are presented for each sub-category. To enhance credibility and validity, the interview guide was meticulously discussed with research and clinical staff. We also ensured that the first author had the required knowledge and training to perform the study.

### Practical implications

Our results clearly indicate the need of developing a solid concept prior to the construction of the planned university inpatient hospice. Herein, the importance of addressing concerns continuously already throughout the planning phase via multi-disciplinary meetings was highlighted and this prospective qualitative study was commended.

## Conclusion

The overall high level of anticipated progress and excitement is very encouraging. Albeit several serious concerns were mentioned, our results indicate that clinical staff and volunteers believe in a highly positive impact and that they are aware of the need for a university inpatient hospice that combines humanitarian aspects with high-tech medicine. A general consensus is the improvement of end-of-life care by expanding its continuum of care using the intended university inpatient hospice.

## Supplementary Information

**Additional file 1.** Semi-structured interview guide.

**Additional file 2.** Supporting quotes.

## Data Availability

The data used and analysed during the current study are available from the corresponding author on reasonable request.
