# Supplementary material for: “Such an institution represents the circle of life” – bringing an inpatient hospice into an academic setting: a pre-implementation exploratory study
Source: BMC Palliat Care. 2023 Jul 19;22:96. doi: 10.1186/s12904-023-01220-6 (PMC10354892; doi:10.1186/s12904-023-01220-6)
Supplement: Supplementary file 1 — Additional file 1. Semi-structured interview guide. [file 12904_2023_1220_MOESM1_ESM.docx]

| **Semi-structured interview guide** | | |
| --- | --- | --- |
|  | | |
| **Introduction** | | |
| You have been invited for participation in this interview because of your outstanding expertise in hospice and / or palliative care. Today I would like to talk to you about your personal opinion on the implementation of the first university inpatient hospice at the [university name] in cooperation with the Department of Palliative Medicine. Herein, I am particularly interested in your expectations and concerns. | | |
|  | | |
| **Transition** | | |
| With this interview, we would like to explore your expectations and concerns of the first university inpatient hospice. The plan for the construction and the location is currently becoming more concrete, but we depend on your help in developing a concept and can thus benefit from your expertise. How do you envision the intended university inpatient hospice at the [university name]? | | |
|  | | |
| **Key questions** | | |
| **Starting questions** | **Probing questions** | |
| **Opportunities and expectations**  In your opinion, what opportunities does the university inpatient hospice at the [university name] offer and what are your expectations regarding its implementation? | - What could be advantageous? - Compared to a regular inpatient hospice and / or palliative care unit, what prospects or differences do you see? - What opportunities might this university inpatient hospice offer for patients or what wishes could patients have for a university inpatient hospice? - What are you looking forward to as a <profession> with respect to the implementation of the university inpatient hospice? | |
| **Challenges and concerns**  What challenges and concerns do you have with respect to the implementation of the proposed university inpatient hospice? | - Which problems do you see and what solutions can you imagine? - What could be particularly difficult to implement? | |
| **Criteria and general requirements**  What are important criteria and general requirements during planning and implementation? | - Did you already list these criteria in order of priority or could you possibly prioritize them? - In your opinion, what are necessary requirements in terms of spatial design and staffing? | |
|  |  |  |
| **End of interview** | | |
| Is there anything else you might want to add? E.g., something that is important to you that has not been addressed yet?  Thank you very much for your participation. This will surely help in developing a concept for the first university inpatient hospice at the [university name]. | | |
