# Supplementary material for: “Such an institution represents the circle of life” – bringing an inpatient hospice into an academic setting: a pre-implementation exploratory study
Source: BMC Palliat Care. 2023 Jul 19;22:96. doi: 10.1186/s12904-023-01220-6 (PMC10354892; doi:10.1186/s12904-023-01220-6)
Supplement: Supplementary file 2 — Additional file 2. Supporting quotes. [file 12904_2023_1220_MOESM2_ESM.docx]

**Supplemental file 2.** Supporting quotes

| Staff: Leadership | |
| --- | --- |
| 1. | For example, it must be led by nurses, i.e., the management position must be filled by a nurse and therewith be autonomous from other sub-units of the department which are led by a medical director. (clinical staff, 01) |
| Staff: Staffing | |
| 2. | There must be a clear separation of staff, that is, that new employees are hired who want to work in the hospice and then only work there and that those who currently work in the palliative care unit do not have to leave and work in the hospice. That would certainly work for a day, but not for the long term. I don’t even know if that’s allowed by the authorities. (clinical staff, 04) |
| Organizational level: Innovation | |
| 3. | Well, in my opinion, the greatest opportunity is the fact that we could include groups of patients into our care, if we wanted to - of course, an appropriate concept would have to be developed for this - which we do not care for in a prototypical hospice. I am thinking primarily of severely affected neurological patients such as patients with amyotrophical lateral sclerosis or multiple sclerosis or Parkinson's disease or whatever. (clinical staff, 03) |
| Organizational level: Expanding and intensifying competencies and services | |
| 4. | I even believe that it feels very good for relatives to get in touch with one another. I think that's a good way to start a conversation and start mourning, simply put - you're not alone. (volunteer, 09) |
| Organizational level: Strengthening synergies and legal requirements | |
| 5. | … and certainly that there is no negative competition between the different departments of the university clinic but that the expertise and scientific knowledge of every department will be accessible to hospice residents. (volunteer, 15) |
| 6. | Then, of course, financing must be completely separate. On the one hand, we are part of a hospital and must generate a profit. On the other hand, an inpatient hospice is not allowed to make a profit but depends on donations. (clinical staff, 06) |
| Organizational level: Equitable access | |
| 7. | … but I think it's important to give people access- access to people who don't have access to palliative care yet. (clinical staff, 01) |
| Organizational level: Public relations | |
| 8. | I think these topics [grief, death, and dying] will become more present at university but also in society, this project might raise people’s interest in these topics, reducing their fear of death, while decreasing the taboo element of death. (clinical staff, 11) |
| Organizational level: Food service | |
| 9. | And for many patients who can still eat, that is the only highlight left, a delicious dish. And I would find it nice (…), that there is someone permanently employed, a cook, who perhaps cannot respond to every wish, but can respond to wishes and can prepare a delicious meal (…) because as I said, that is the last true feeling of joy for many. (volunteer, 09) |
| Organizational level: Financing | |
| 10. | (…) only the financial aspect, that patients do not care for. That the palliative care ward is financed by the health insurance and the hospice is financed by the health insurance and long-term care insurance, that of course, is of very little interest, very little. (clinical staff, 13) |
| Organizational level: Rejection of hospice movement | |
| 11. | Another fear is, on the other side, what I have just mentioned, that the hospice movement is like "For God's sake, now the university medicine also wants to take possession of the hospice movement” and might therefore reject it completely without thinking about it any further. (clinical staff, 06) |
| Organizational level: Structural factors / hospice character | |
| 12. | I'm a bit afraid that it will be too medical and not cozy enough, which usually characterizes a hospice, more peace and quiet, little hustle and bustle, that it's just like home. (clinical staff, 07) |
| Patient-centered care: Continuity of care | |
| 13. | And that is a holistic approach, to say that the combination of palliative medicine and hospice allow for a qualitative improvement at the end of life, I think that is extremely important for patients and their relatives because it is stressing them out, once they have gotten used to the concept of palliative care having to leave and where to receive hospice care. (clinical staff, 11) |
| Patient-centered care: Patient understanding | |
| 14. | Let's assume that we refer a patient from another department to the palliative care ward (…) if it were not spatially separate from the hospice, then I wonder whether that patient understands (…), feels, sees that this [hospice] is yet another place. (clinical staff, 12) |
| Patient-centered care: Humanity | |
| 15. | Humanity should really be the highest priority, humanity and real dignity, which is being said over and over again, yes, one should - everyone wishes to die in dignity. (volunteer, 15) |
